# Brain Tumor/Mass Classification Framework Using Magnetic-Resonance-Imaging-Based Isolated and Developed Transfer Deep-Learning Model

**DOI:** 10.3390/s22010372

**Published:** 2022-01-04

**Authors:** Muhannad Faleh Alanazi, Muhammad Umair Ali, Shaik Javeed Hussain, Amad Zafar, Mohammed Mohatram, Muhammad Irfan, Raed AlRuwaili, Mubarak Alruwaili, Naif H. Ali, Anas Mohammad Albarrak

**Affiliations:** 1 Radiology, Department of Internal Medicine, College of Medicine, Jouf University, Sakaka 72388, Saudi Arabia; mfalanazi@ju.edu.sa (M.F.A.); Raalruwaili@ju.edu.sa (R.A.); msmalruwaili@ju.edu.sa (M.A.); 2 Department of Unmanned Vehicle Engineering, Sejong University, Seoul 05006, Korea; umair@sejong.ac.kr; 3 Department of Electrical and Electronics, Global College of Engineering and Technology, Muscat 112, Oman; m.mohatram@gcet.edu.om; 4 Department of Electrical Engineering, The Ibadat International University, Islamabad 54590, Pakistan; 5 Electrical Engineering Department, College of Engineering, Najran University, Najran 61441, Saudi Arabia; miditta@nu.edu.sa; 6 Department of Internal Medicine, Medical College, Najran University, Najran 61441, Saudi Arabia; dr.naif1989@gmail.com; 7 Department of Internal Medicine, College of Medicine, Prince Sattam Bin Abdulaziz University, Alkharj 16278, Saudi Arabia; a.albarrak@psau.edu.sa

**Keywords:** brain tumor, brain mass, brain MRI images, deep-learning model, tumor classification

## Abstract

With the advancement in technology, machine learning can be applied to diagnose the mass/tumor in the brain using magnetic resonance imaging (MRI). This work proposes a novel developed transfer deep-learning model for the early diagnosis of brain tumors into their subclasses, such as pituitary, meningioma, and glioma. First, various layers of isolated convolutional-neural-network (CNN) models are built from scratch to check their performances for brain MRI images. Then, the 22-layer, binary-classification (tumor or no tumor) isolated-CNN model is re-utilized to re-adjust the neurons’ weights for classifying brain MRI images into tumor subclasses using the transfer-learning concept. As a result, the developed transfer-learned model has a high accuracy of 95.75% for the MRI images of the same MRI machine. Furthermore, the developed transfer-learned model has also been tested using the brain MRI images of another machine to validate its adaptability, general capability, and reliability for real-time application in the future. The results showed that the proposed model has a high accuracy of 96.89% for an unseen brain MRI dataset. Thus, the proposed deep-learning framework can help doctors and radiologists diagnose brain tumors early.

## 1. Introduction

The brain is one of the most complex and immense parts/organs of the human body, having more than 100 billion nerve cells, which have trillions of connections known as synapses [1]. The human brain works as the nervous system’s central command/control center to regulate the whole-body organs. Therefore, the existence of any abnormality in the brain has a fatal impact on human health conditions. For example, in 2020, almost 10 million deaths were reported due to cancer, the second leading cause of death worldwide, according to the World Health Organization (WHO) [2]. Therefore, the early detection of cancer increases the patient’s survival chances. However, unlike cancer, a brain tumor is an abnormal, uncontrolled, and unnatural growth of the human brain cell.

The brain tumor can be classified as benign or malignant depending on its position, progression stage, nature, and growth rate [3,4]. In the case of a benign brain tumor, the affective cells rarely attack the nearby healthy cells. It also has a sluggish progression rate and distinct boundaries such as pituitary and meningioma. Whereas in malignant brain tumors, the affective cells impact the neighboring healthy cells (spinal cord or brain) and have a high progression rate with vast boundaries such as glioma. Depending upon the origin, the brain tumor can further be categorized as a primary or secondary brain tumor [5]. If the tumor originates in the brain tissues, it classifies as a primary tumor. If the tumor exists in other parts of the body and enters the brain through blood vessels, it can be classified as a secondary tumor.

According to the WHO, brain tumors can further be characterized depending upon their boundary, severity, and growth rate [6,7,8]. At stage 0, the cancerous affected tumor cells are bounded and have no impact on nearby healthy cells. The cancerous affected tumor cells begin to affect the neighboring healthy cell in stages 1, 2, and 3. In the final stage (stage 4), cancer affects all bodies, and it is almost impossible to save human life. Therefore, for cancer treatment, early-stage detection and differentiation of cancer (meningioma, pituitary, and glioma) are essential to save the patient’s life.

Various diagnostic approaches, invasive and non-invasive, are used to detect cancer in the human brain [9]. For example, in a biopsy, which is an invasive approach, a sample is collected by incision and observed by pathologists using a microscope to check its malignancy. However, unlike tumors in other parts of the body, a brain-tumor biopsy is not usually done before definitive brain surgery. Therefore, non-invasive imaging approaches such as magnetic resonance imaging (MRI), positron emission tomography, and computed tomography are considered rapid and safer techniques for brain-tumor diagnosis than biopsy. In all aforementioned non-invasive imaging modalities, the MRI is the most preferred because of its detailed information about the brain tumor’s location, progression, shape, and size in 2D and 3D formats [10]. However, manually interpreting the MRI image is time consuming for medical practitioners and has a high chance of error due to a large number of patients.

With the advancement of intelligent learning algorithms, the efficiency of computer-aided-diagnosis (CAD) systems has improved to assist the doctor in diagnosing brain tumors [5,11,12]. Various approaches have been reported to diagnose brain tumors using traditional/classical machine-learning and deep-learning methods [13]. In classical machine-learning approaches, classification accuracy mainly relies on extracting the most related features. The feature extraction can be categorized as global (low)- and local (high)-level features. In global-level features, the texture, first-order, and second-order statistics features are used to train the classical classifier such as support vector machine (SVM), Naïve Bayes, tree, etc. In a study [14], the gray-level co-occurrence matrix was used to train the SVM model to classify the brain MRI images into binary classes (normal and abnormal). Their trained model had reasonably high accuracy, but the training time was high. In the consequent study [15], the principal component analysis was utilized to decrease training time by reducing the training features dimensions. In multiclass classification, the accuracy of the global-feature-trained model is low because of the same appearance (size, intensity, texture, etc.) of brain tumor types. Local-level features such as scale-invariant feature transformation [16], fisher vector [17], and a bag of words [18] have also been employed to address this issue. The accuracy of these approaches mainly relies on prior information about the position or location of the tumor in brain MRI images, which increases the chance of errors.

With the evolution of machine-learning algorithms in the last few years, deep-learning algorithms can automatically compute the optimal data features. Deep-learning networks, such as convolutional neural networks (CNN) and fully convolutional networks, are widely applied to classify MRI images in order to diagnose brain tumors [19]. The classification of brain images using a CNN can be done using a pre-trained network and a designed network (designed by various researchers). Pereira et al. [20] designed a CNN to classify the brain mask and whole-brain images into binary classes. Their proposed model had an accuracy of 89.5% and 92.9% for the whole brain image and brain mask, respectively. In 2019 [21], the simplest form of a CNN was proposed to classify brain images into three classes (glioma, meningioma, and pituitary), and a classification accuracy of 84.19% was reported.

Furthermore, a multiscale, 3D deep CNN was proposed to classify images into subclasses of glioma (low- and high-grade glioma). The proposed model achieved a high accuracy of 96.49%. In a recent study [9], a 22-layer network was trained to classify brain images into three classes. They utilized an online MRI brain-image dataset to validate their proposed model [22]. In addition, they utilized the data-augmentation approach to increase the size of the dataset by three times (3064 was the original size) for better training of the model. They also used a 10-fold cross-validation approach for the model’s training and had an accuracy of 96.56%. In a recent study [23], two different CNN models of 13 and 25 layers were proposed to classify brain images into two and five classes, respectively. With the increase in classes, the accuracy of the proposed model dropped to 92.66%. The use of two different models for the detection and differentiation of the brain tumor was also a shortcoming of the approach. Deepak et al. [24] utilized a pre-trained (GoogleNet) network to classify brain images into three classes. They reported a high accuracy of 98% for an online dataset. Furthermore, the accuracy of various pre-trained networks using the transfer-learning approach was also checked using the brain MRI dataset [25]. The authors found that the ResNet-50 had a high classification accuracy of 97.2% for the binary problem against a small dataset of brain images. However, the training time of the pre-trained network was very high. To tackle this issue, Kang et al. [26] computed the feature of brain images using pre-trained networks and trained the classical classifier. They found that the ensemble features computed using DenseNet-169, ShuffleNet V2, and MnasNet with the SVM had the best testing accuracy of 93.72% for four classes (no tumor, glioma, meningioma, and pituitary). They also utilized the data-augmentation technique to validate the model, which resulted in a higher accuracy. The literature has shown that data augmentation helps to enhance the classification accuracy; however, its reliability is still not proven for real-time application. Therefore, further research is needed to detect and differentiate the brain tumor. In addition, the accuracy of the trained model must be checked against the MRI brain images of another machine (whose images are not used for training).

Driven by the desire to increase the reliability, true grading, early diagnostic, and accurate classification of brain tumors, this work proposes an automatic detection and differentiation CNN model for brain MRI images. In this work, an isolated, 22-layer-based CNN is modeled from scratch to group the brain MRI images into binary classes (tumor and non-tumor). Furthermore, to differentiate between the various types of tumors such as glioma, meningioma, and pituitary, the modeled 22-layer isolated CNN is re-utilized using the transfer-learning approach. Finally, the various online MRI brain datasets are used to check and compare the proposed approach’s performance. The result of the proposed model is compared with other networks found in the literature that did not use a data-augmentation approach. Another machine’s brain images also fed the trained model in order to verify the accuracy, adaptability, and reliability of the developed transfer-learned model.

## 2. Materials and Methods

### 2.1. Magnetic Resonance Imaging (MRI) Dataset

In this work, three different online datasets of brain MRI images were utilized. The first publicly available dataset of binary-class brain MRI images was downloaded from the Kaggle website; for the sake of simplicity, this data was named dataset-I in this work [27]. This dataset contained a total of 3000 brain MRI images of tumor and no-tumor classes (1500 for each class). The other dataset used in this study was also downloaded from the Kaggle website [28]; it contained 826, 822, 395, and 827 brain MRI images of glioma tumor, meningioma tumor, no tumor, and pituitary tumor, respectively. For simplicity, this data was named dataset-II in this work. Finally, another dataset of 233 patients’ brain MRI images was used in this study [22]. These brain MRI images were collected at two hospitals in China (Nanfang Hospital and General Hospital). It had 3064 brain MRI images (1426 glioma tumors, 708 meningioma tumors, and 930 pituitary tumors); this dataset was named a dataset-III in this work. Each type of brain MRI image from all datasets is shown in Table 1.

### 2.2. Isolated and Transfer Learning

An isolated deep-learning network is a network that learns a task from scratch and does not require any previously learned knowledge [29]. However, in the case of transfer learning, the new build model utilizes the previously learned knowledge of other networks [30]. In other words, in transfer learning, a base model is trained using the base images for other tasks, and its learned features are reused for the training of the required model for the targeted task [31]. The two most commonly used methods for transfer learning are pre-trained networks and the new develop-model methods [31]. In the pre-trained-network approach, various publicly available models such as ResNet50, ShuffleNet, GoogleNet, MobileNet v2, SqueezeNet, Inception V3, etc., trained by other researchers, can be reused through transfer learning for the specific task. Whereas in the develop-model method, a new model is developed from scratch, and neurons’ weights are re-utilized by changing some of the specific layers of the CNN model for the targeted task. In this work, an isolated CNN was trained from scratch using the dataset-I of brain MRI images, and then it was re-utilized to train the deep-learning model through transfer learning for dataset-II. The design of the isolated CNN is discussed in the next section.

### 2.3. Methodology

This section explains the proposed approach for modeling an isolated network from scratch and the developed transfer deep-learning model.

#### 2.3.1. Magnetic Resonance Images Pre-Processing

All brain MRI images contain undesired information in the form of noise, which leads to low classification accuracy, also discussed by Kang et al. [26]. Therefore, it is necessary to remove the noise and the undesired areas to yield useful information. The extreme point is calculated using the cropping method; the erosions and dilation operation were applied to remove the noise; further detail about this method can be found in [26,32]. The width, height, and sizes of all brain images in the MRI datasets were not the same; all the images were resized to 227 × 227 in order to attain uniformity. Then, all the images were encoded into the range of 0–255. Finally, all the images were also normalized/scaled before being fed to the model.

#### 2.3.2. Developed Isolated and Transfer Deep-Learning Models

As discussed earlier, two machine-learning (isolated CNN and transfer learning) approaches were investigated in detail. First, the various architectures of isolated CNN were modeled (19, 22, and 25 layers) to evaluate their performance in classifying brain MRI images into 2, 3, and 4 classes. Then, after evaluation, the best isolated-CNN model was re-utilized by using the transfer-learning approach. The architecture and parameters of the models are discussed in complete detail in the subsequent sections.

##### Isolated Convolutional Neural Network Model

**Figure 1:**
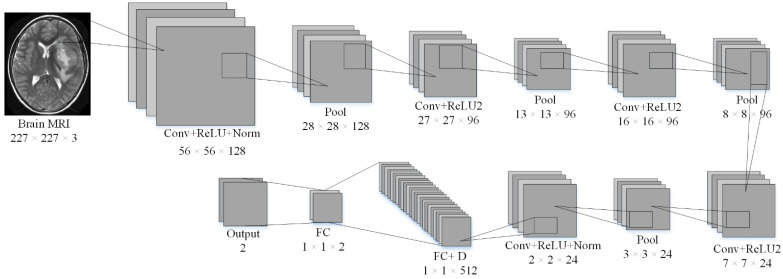
The architecture of the isolated-CNN model was built from scratch.

A typical CNN can easily be divided into two main parts: extraction of features and classification/prediction. The general architecture of the CNN models has five main layers (input, convolutional, pooling, fully connected, and classification). The convolutional and pooling layers are used to extract the features, whereas the fully connected layers and classification layers are used for prediction/classification. In this work, an isolated CNN was developed from scratch to classify brain MRI images into different classes. Isolated-CNN models with 19 layers, 22 layers, and 25 layers were built to check the accuracy of dataset-I and dataset-II. The input layer of the isolated-CNN model consisted of the brain images’ pixel value. The 22-layer isolated CNN had the best accuracy for the classification of both datasets. The details about the parameters and structure of the 22-layer isolated CNN for binary classification are given in Table 2 and Figure 1.

##### Transfer Learning

**Figure 2:**
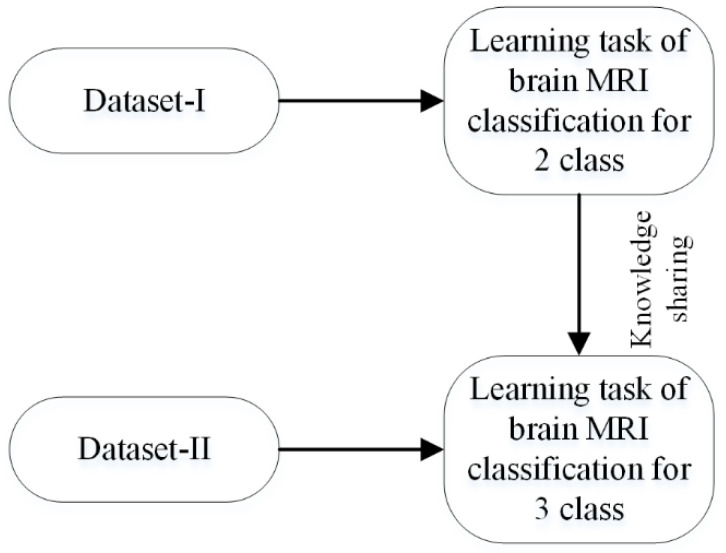
Process of transfer learning for brain-image classification.

This work used a new develop-model method for transfer learning, as discussed in Section 2.2. First of all, an isolated network discussed in the previous section was trained for binary classification using the MRI dataset-I [27]. The trained model (can be named as a pre-trained 2-class model) was then fine-tuned on the brain MRI dataset-II [33]. In this approach, not only was the final layer of the pre-trained model replaced, but some of the previous layers can also be re-trained. After comprehensive training, it was found that the impact of fine-tuning on the new transfer-learned network (learned network) was almost negligible. The diagram of the transfer-learning approach is shown in Figure 2.

##### Optimization

**Figure 3:**
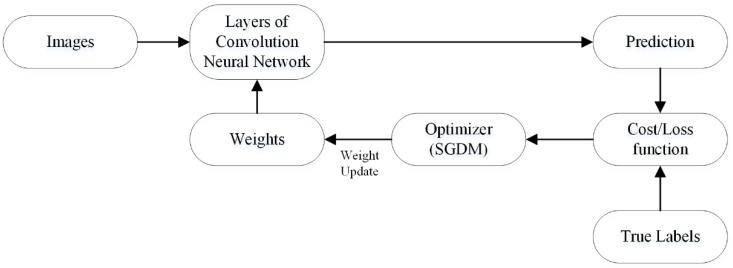
The weights update/learning process of convolutional neural network.

In deep learning, optimization decreases the cost/loss function’s value in order to enhance model accuracy. In other words, optimization measures the learning process’s progress by computing the learnable parameter resulting in loss reduction. In CNN architecture, the convolutional layer filters have learnable parameters to compute features of the image. In the training process, parameters are randomly initialized, and the loss is computed in each epoch based upon the prediction and targeted labels. Subsequently, in the next epoch, the optimizer updates learnable parameters, and this process continually updates the parameters to find the minimal loss value. The working process of the optimizer is shown in Figure 3.

The stochastic gradient descent with momentum (SGDM) was used for optimization in this work. The initial rate, epochs, and momentum values were 0.001, 100, and 0.9, respectively.

#### 2.3.3. Proposed Framework

**Figure 4:**
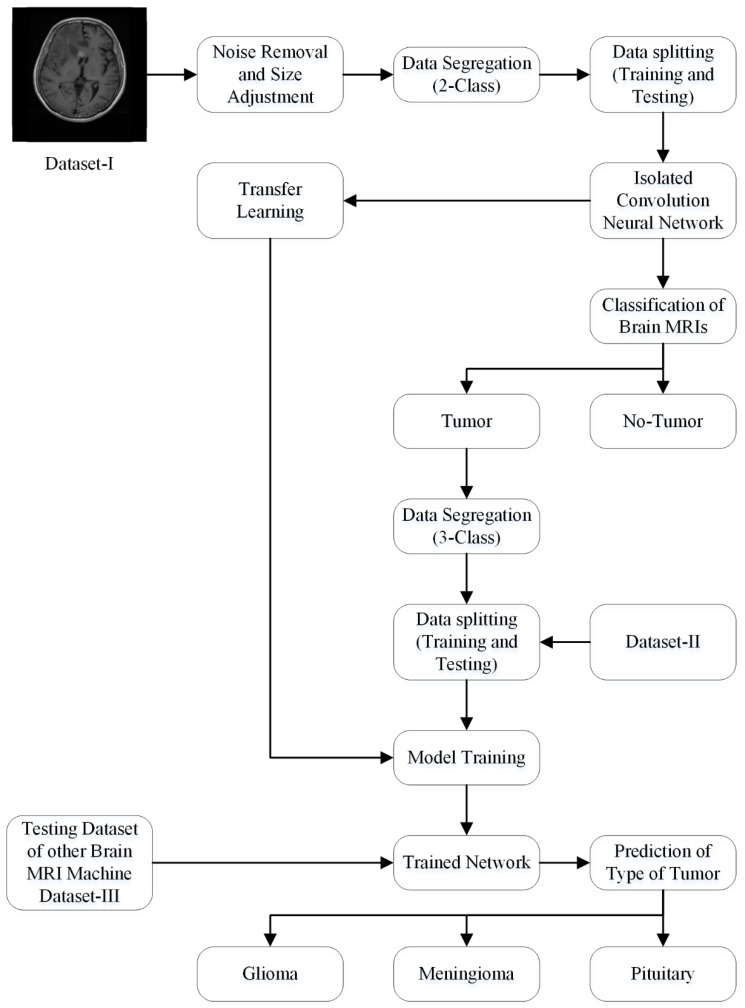
The framework of the proposed approach.

The generic structure of the isolated CNN that was developed from scratch is shown in Figure 1. Dataset-I and II were utilized for the training and testing of the 19-, 22-, and 25-layer isolated-CNN models. The 22-layer isolated CNN had the best accuracy for classifying brain MRI images into tumor and non-tumor class using dataset-I. The images of the non-tumor class of dataset-II were also used to train the binary-class isolated CNN. Finally, the pre-trained 2-class model was re-utilized using the transfer-learning method in order to re-adjust the weights of neurons to categorize the tumors into subclasses (glioma tumor, meningioma tumor, and pituitary tumor) for various tumor images of dataset-II. The complete framework of the proposed approach is shown in Figure 4.

The datasets I and II images were randomly distributed into the training and testing sets at the ratio 80 and 20%, respectively, to check the performance of the networks. For a fair comparison, all the parameters of training and validation were kept constant for each network.

## 3. Results

In this work, MATLAB 2021a running on a personal computer having a specification of Core i7, 7th Generation, 16 GB RAM, NVIDIA GeForce 1060, 1 TB SSD, and 64-bit Windows 10 Education operating system was utilized to run all the simulations and perform all the analyses. In addition, both the datasets (dataset I and II) were randomly divided into two groups of training (80% of all images in the dataset) and testing (20% of all images in the dataset) to avoid overfitting.

Various isolated-CNN models were built by changing their architecture and parameters to check their performances for both datasets. Finally, all the isolated-CNN models were compared using validation accuracy, validation loss, training accuracy, training loss, and training time. The results of the 19-layer, 22-layer, and 25-layer CNN models for two- and four-class classification are shown in Table 3 and Table 4.

**Figure 5:**
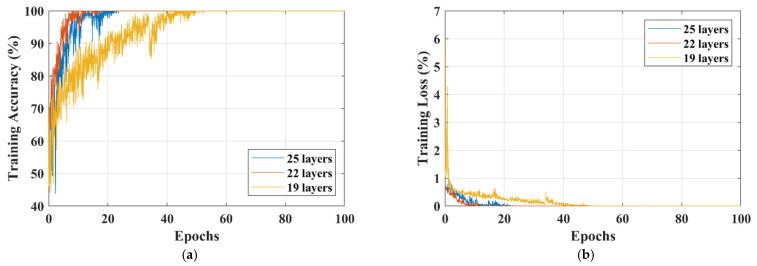

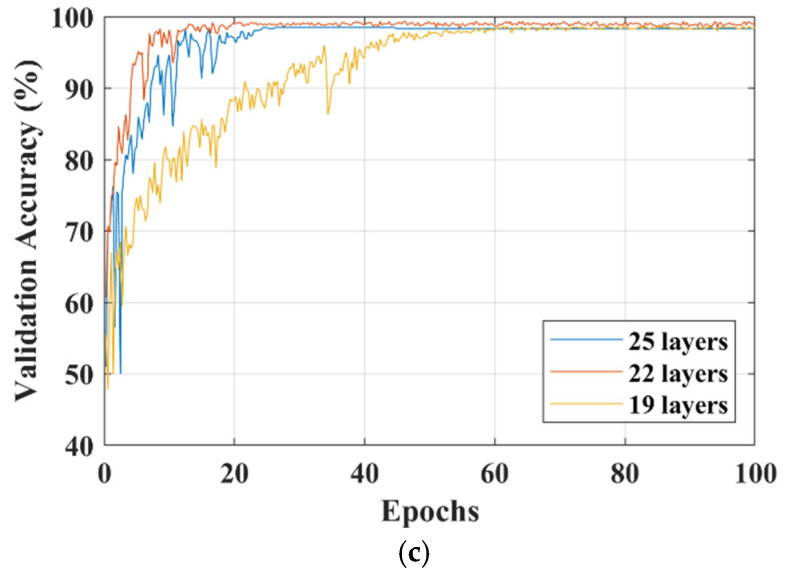
Comparison of different isolated-CNN models for dataset-I; (**a**) Training-accuracy curves; (**b**) Training-loss curves; (**c**) Validation-accuracy curves.

**Figure 6:**
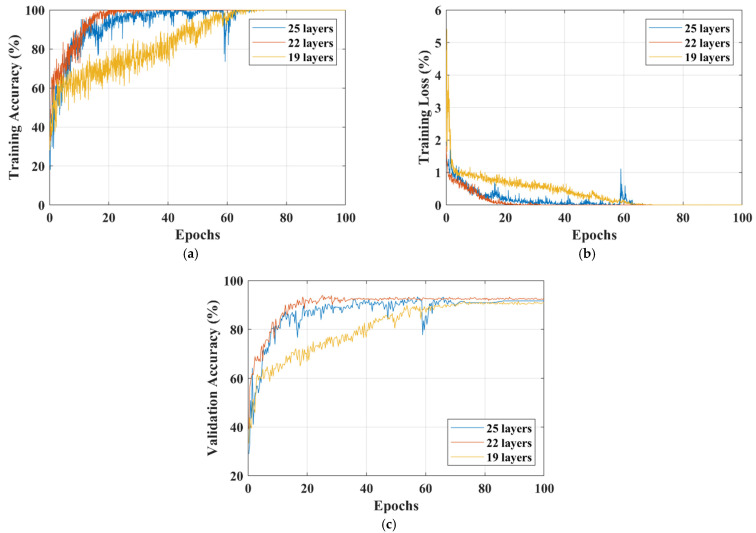
Comparison of different isolated-CNN models for dataset-II; (**a**) Training-accuracy curves; (**b**) Training-loss curves; (**c**) Validation-accuracy curves.

The training accuracy, training loss, and validation accuracy curves of each isolated-CNN model for dataset-I and dataset-II are shown in Figure 5 and Figure 6, respectively.

After deeply analyzing the results of the 19-, 22-, and 25-layer isolated CNNs, it was found that the 22-layer isolated-CNN model had the best classification accuracy for dataset-I and dataset-II (see Table 3 and Table 4). It is also important to note that the 22-layer isolated CNN reached 98% training accuracy in 9 epochs for binary classification, whereas it took only 17 epochs to train the model for dataset-II (see Figure 5 and Figure 6). Figure 5c and Figure 6c also show the isolated-CNN model’s high testing/validation accuracy for datasets-I and II, respectively. The classification accuracy of detecting the tumor in the human brain using the 22-layer isolated CNN was very high (99.33%). However, the accuracy of differentiating between the various types was just 92.67%.

**Figure 7:**
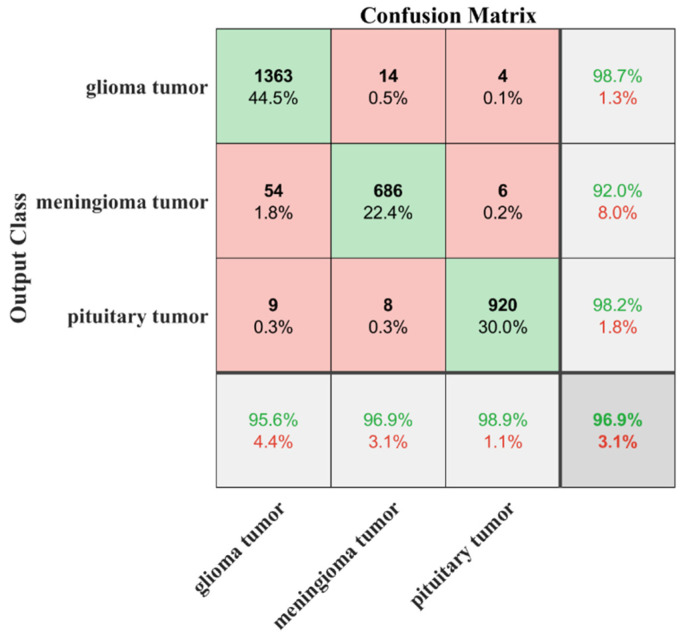
Results of testing of developed transfer-learned model for dataset-III.

Therefore, with the desire to increase the differentiation accuracy, the isolated CNN trained with binary-class images was re-utilized by using the transfer-learning method to train the model using various types of brain images in dataset-II, as discussed in Section 2.3. Dataset-II contained 826, 822, and 827 brain MRI images of glioma tumors, meningioma tumors, and pituitary tumors, respectively. Before starting the training process, the dataset-II images were separated at an 80:20 ratio for the training and testing sets, respectively, to avoid overfitting. The result of the training accuracy of the proposed transfer-learned model is shown in Figure 7. The proposed model reached a training accuracy of 98.5% in just nine epochs. The results of the testing of the proposed model are listed in Table 5.

**Figure 8:**
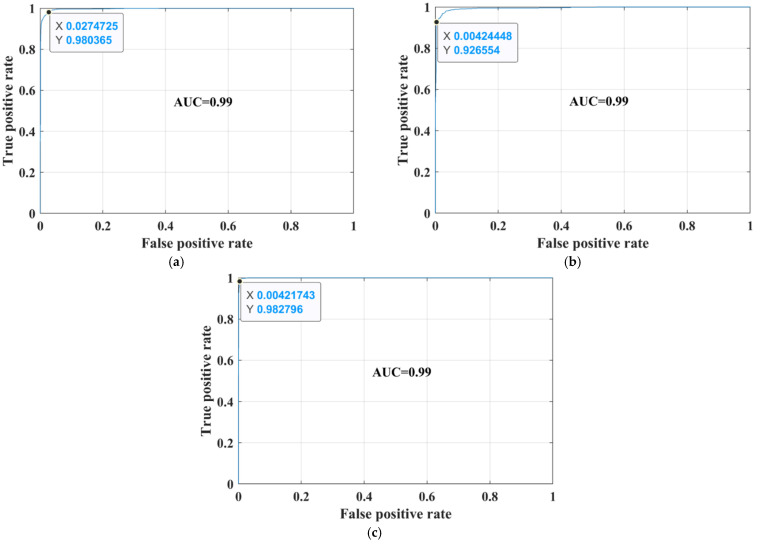
Receiver operation characteristic (ROC) curves for dataset-III; (**a**) glioma; (**b**) meningioma; (**c**) pituitary.

The sensitivity/true-positive rate (TPR), miss/false-negative rate (FNR), precision/positive predictive value (PPV), and false-discovery rate (FDR) were used as accuracy-measurement metrics. The proposed developed transfer-learned model had a high TPR of 96.32%, 94.97%, and 95.93% for glioma, meningioma, and pituitary classes, respectively. In addition, the PPV of the pituitary class was 100%, which means that no image of another class was falsely categorized as a pituitary class. Thus, the overall noted accuracy of the proposed developed transfer-learned model was 95.75%. To further validate the proposed model’s adoptability for another machine’s brains MRI images, the developed transfer-learned model was tested using dataset-III. The confusion matrix and AUC curves of the testing of dataset-III are presented in Figure 7 and Figure 8. Table 6 shows the performance comparison of the proposed model with the literature.

## 4. Discussion

Recently, the use of the CNN model to diagnose medical diseases has exponentially increased using medical imaging. Various researchers have presented different training approaches for classifying brain MRI images [9,17,23,26,34,35,36,37,38].

Cheng et al. [39] presented a tumor region augmentation and partition approach to enhance the classification accuracy. Irmak. [23] presented three different CNN models: (i) detection of the tumor (tumor or no tumor); (ii) the type of tumor; and (iii) the stage of the tumor. The reported testing accuracy in classifying the tumor into types was 92.66%. In another study [9], the authors trained the network using the 3-class brain MRI images (dataset-III); they performed a 10-fold cross-validation method to train the model. They reported the best classification accuracy of 95.40% for the original dataset. They also checked the accuracy of their approach for the augmented images dataset (96.56% accuracy). Sultan et al. [35] proposed a CNN model for dataset-III; their framework took almost 5 h to train the model and had an accuracy of 96.13%. Rehman et al. [34] utilized the pre-trained network to classify the images into their respective categories. The accuracy of pre-trained models such as AlexNet, GoogleNet, and VGGNet was checked using the fine-tune approach. The accuracies of 95.86%, 95.61%, and 95.42% with a training time of 42 min 36 s, 79 min 25 s, and 89 min 30 s was reported for AlexNet, GoogleNet, and VGGNet fine-tuned models, respectively, for the original datasets.

This work proposes a transfer-learning model approach to classify brain MRI images to address high training time and adaptability issues. Firstly, the proposed approach classifies the brain MRI images into two classes (tumor or no tumor) with a high accuracy of 99.33% and less training time (see Table 3). Then, the advantage of the transfer-learning approach is utilized to classify the brain MRI images into tumor subclasses. Finally, the proposed approach classifies the brain-tumor images into further categories with an accuracy of 95.75% and a minimal training time of almost 13 min, proving the proposed approach’s robustness and high detection rate (see Table 5). After comparing the proposed approach with the literature in Table 6, it is found that the architecture of the pre-trained model is complex compared to the proposed network [34]. Kang et al. [26] computed the deep features to train the SVM model. The training-vector feature size was large and required a high computational time for training. Furthermore, in the proposed approach, a new dataset-III (which is not used for training and images are collected from the other machine) is utilized to check the adaptability of the proposed approach. The classification accuracy of 96.9% is noted for an unseen dataset using the proposed framework (see Figure 7).

According to the author’s best knowledge, the presented approach is the first approach that validates its adaptability for different MRI brain-imaging machine datasets (i.e., dataset-III) using transfer learning. Furthermore, the proposed framework is very simple and can be helpful for real-time-diagnosis applications in the future. Therefore, the proposed approach can play a pivotal role in helping doctors and radiologists with the early diagnostic of brain tumors.

## 5. Conclusions

This study analyzed the classification accuracy of various isolated-CNN models against various brain MRI datasets. The 22-layer, binary-classification (tumor or no tumor) isolated-CNN model was re-utilized to train the CNN model using the transfer deep-learning concept to identify the tumor subclass. Dataset-I was utilized for training the isolated-CNN model that was built from scratch for binary classification and had an accuracy of 99.33%. Dataset-II was utilized for training the developed transfer-learned model to detect the subclass of the tumor. The developed transfer-learned model showed a high testing accuracy of 95.75% for the brain MRI images of dataset-II, which were not used for training. Furthermore, an unseen dataset-III of a different MRI machine was fed to the developed transfer-learned model to check its accuracy. The proposed model accurately classified 2969 MRI brain images out of 3064 with a high classification accuracy of 96.9%. The robustness, adaptability, generalization capability, and high accuracy make the proposed framework helpful to use in real-time diagnosis applications in future.

## Figures and Tables

**Table 1:** Classification of brain MRI images.

	No Tumor	Glioma Tumor	Meningioma Tumor	Pituitary Tumor
Brain MRI Images	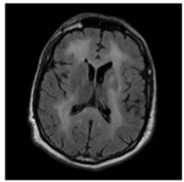	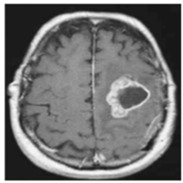	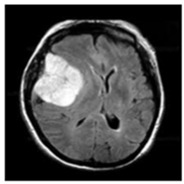	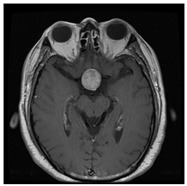

**Table 2:** Information related to the 22-layer CNN developed from scratch.

Layer No.	Layer Type	Properties	Learnable
1	Image Input	227 × 227 × 3 images with ‘zerocenter’ normalization	-
2	Convolutional	128 6 × 6 convolutions with stride [4 4] and padding [0 0 0 0]	Weights: 6 × 6 × 3 × 128 Bias: 1 × 1 × 128
3	ReLU	ReLU	-
4	Cross Channel Normalization	cross channel normalization with 5 channels per element	-
5	Max	2 × 2 max pooling with stride [2 2] and padding [0 0 0 0]	-
6	Convolutional	96 6 × 6 convolutions with stride [1 1] and padding [2 2 2 2]	Weights: 6 × 6 × 3 × 128 × 96 Bias: 1 × 1 × 96
7	ReLU	ReLU	-
8	Max	2 × 2 max pooling with stride [2 2] and padding [0 0 0 0]	-
9	Convolutional	96 2 × 2 convolutions with stride [1 1] and padding [2 2 2 2]	Weights: 2 × 2 × 96 × 96 Bias: 1 × 1 × 96
10	ReLU	ReLU	-
11	Max	2 × 2 max pooling with stride [2 2] and padding [0 0 0 0]	-
12	Convolutional	24 6 × 6 convolutions with stride [1 1] and padding [2 2 2 2]	Weights: 6 × 6 × 96 × 24 Bias: 1 × 1 × 24
13	ReLU	ReLU	-
14	Max	2 × 2 max pooling with stride [2 2] and padding [0 0 0 0]	-
15	Convolutional	24 6 × 6 convolutions with stride [1 1] and padding [2 2 2 2]	Weights: 2 × 2 × 24 × 24 Bias: 1 × 1 × 24
16	ReLU	ReLU	-
17	Batch Normalization	Batch normalization	Offset: 1 × 1 × 24 Scale: 1 × 1 × 24
18	Fully	512 fully connected layer	Weights: 512 × 96 Bias: 512 × 1
19	Dropout	30% dropout	-
20	Fully	2 fully connected layer	Weights: 2 × 512 Bias: 2 × 512
21	Softmax	-	-
22	Classification Output	-	-

**Table 3:** Comparison of isolated-CNN models for binary-class classification (tumor and no tumor) using dataset I.

Network	Training Accuracy (%)	Training Loss	Training Time	Validation Accuracy (%)	Validation Loss
19-layers	100	2.8016 × 10^−5^	15 min 58 s	98.50	0.0850
22-layers	100	4.8811 × 10^−6^	16 min	99.33	0.0534
25-layers	100	4.8243 × 10^−7^	15 min 43 s	98.33	0.1412

**Table 4:** Comparison of isolated-CNN models for four-class classification using dataset II.

Network	Training Accuracy (%)	Training Loss	Training Time	Validation Accuracy (%)	Validation Loss
19-layers	100	1.9454 × 10^−4^	14 min 57 s	91.27	0.4637
22-layers	100	2.7508 × 10^−5^	14 min 35 s	92.67	0.3208
25-layers	100	1.6904 × 10^−6^	14 min 22 s	91.62	0.7276

**Table 5:** Detection of type of brain tumor using developed transfer-learned network.

Class	Classified as			TPR (%)	FNR (%)	PPV (%)	FDR (%)	Training Time	Validation Accuracy
	Glioma	Meningioma	Pituitary						
**Glioma**	157	6	0	96.32	3.68	95.15	4.85	13 min 8 s	95.75%
**Meningioma**	8	151	0	94.97	5.03	92.07	7.93		
**Pituitary**	0	7	165	95.93	4.07	100	0		

**Table 6:** Performance comparison of the proposed model with literature.

Study	Type of Dataset	Model	Accuracy (%)	Training Time
Abiwinanda et al. [21]	Dataset-III	13-layer CNN	84.19	-
Irmak. [23]	Dataset-II	25-layer CNN	92.66	-
Kang et al. [26]	Dataset-III	Pre-trained CNN models with machine-learning classifiers	93.72	-
Rehman et al. [34]	Dataset-III	AlexNet GoogleNet VGGNet	95.86 95.61 95.42	43 min 79 min 89 min
Proposed	Dataset-II Dataset-III	Developed transfer-learned CNN	95.75 96.90	13 min

## Data Availability

Not applicable.
